# The utility of remdesivir in SARS-CoV-2: A single tertiary care center experience from a developing country

**DOI:** 10.1016/j.rcsop.2022.100107

**Published:** 2022-02-08

**Authors:** Muhammad Irfan Malik, Sardar Al Fareed Zafar, Muna Malik, Fabiha Qayyum, Iqra Akram, Ammarah Arshad, Khalid Waheed, Jodat Saleem, Abdul Jabbar, Muhammad Junaid Tahir, Zohaib Yousaf

**Affiliations:** a Postgraduate Medical Institute, Lahore, Pakistan; b Lahore General Hospital, Lahore, Pakistan; c Ameer-ud-din Medical College, Lahore, Pakistan; d Department of Clinical Medicine, Faculty of Veterinary Science, University of Veterinary and Animal Sciences, 54600 Lahore, Punjab, Pakistan; e Hamad Medical Corporation, Doha, Qatar

**Keywords:** Remdesivir, COVID-19, Drug safety, Infection, Treatment, Pakistan

## Abstract

**Background:**

Remdesivir is a monophosphoramidate prodrug of an adenosine analog, and it has a broad-spectrum antiviral activity against paramyxoviruses, flaviviruses, and coronaviruses. Remdesivir is associated with decreased hospital stay and improved outcomes in coronavirus- disease 2019 (COVID-19).

**Methodology:**

Of 846 suspected COVID-19 patients admitted to the hospital, 612 SARS-CoV-2 nasopharyngeal RT-PCR positive patients were evaluated for enrollment in this prospective cohort study. 159 RT-PCR positive patients were given remdesivir. Their clinical, biochemical parameters, hospital stay, and outcomes related to morbidity and mortality were followed.

**Results:**

Out of the 159 patients, 141 recovered after remdesivir use. The Chi-square test for independence examined the relation between the day of the first dose, dose of remdesivir, and clinical outcome. The standardized case fatality ratio (CFR) in the 453 hospitalized patients who did not receive remdesivir was 32.89% (*N* = 149) as compared to 11.32% (*N* = 18) in the patients who received remdesivir. These findings are in keeping with the therapeutic value of remdesivir in symptomatic SARS-CoV-2 infection of varying severity.

**Conclusion:**

The use of remdesivir is associated with a decrease in the severity of the SARS-CoV-2 infection. Its use is also associated with a decreased length of hospital stay and lower mortality than the patients who did not receive remdesivir.

## Highlights

• In this study remdesivir was used as adjuvant therapy in moderate COVID-19 illness.
• Earlier administration of remdesivir led to a lower mortality rate and a shorter duration of hospital stay.
• Inflammatory parameters improved by 7th day of treatment.
• There is a reduction in case fatality ratio with use of remdesivir in moderate COVID-19.

## 1 Introduction

In December 2019, the first case of severe acute respiratory coronavirus 2 (SARS-CoV-2) was reported, that later resulted in one of the largest pandemics in the recent history.^1^^,^^2^ Clinical presentation varies from asymptomatic to life-threatening pneumonia, leading to acute respiratory failure, multiorgan failure, and death.^3^ The morbidity and mortality associated with this novel virus have piqued clinicians' and researchers' interest especially in the potential primary prevention and possible effective treatments.^4^ Reducing the severity of the disease, in-hospital stay, and improving the disease-associated morbidity and mortality are the other areas of interest for this novel virus. Since the start of the pandemic, there is a renewed focus on repurposing existing medications and therapies for the treatment of SARS-CoV-2. Biotherapeutics are an essential part of this global effort.^5^

SARS-CoV-2 shares up to 80% genetic homology with other Coronaviruses like the SARS-CoV-1 and up to 50% with the MERS-CoV.^6^ This homology gives rise to a possibility of using similarity in management strategies, including drugs used for the management of other coronavirus associated diseases.^7^ During the initial wave of the pandemic, experts in China and Pakistan recommended herbal products for treating coronavirus- disease 2019 (COVID-19).^8^^,^^9^ However, to date, evidence, including randomized controlled trials, is lacking.^10^

Remdesivir is a monophosphoramidate prodrug of an adenosine analog, and it has a broad-spectrum antiviral activity against paramyxoviruses, flaviviruses, and coronaviruses.^11^ The effectiveness of remdesivir has been reported against different groups of coronaviruses, including Alphacoronavirus NL63 and several SARS/MERS-CoV coronaviruses. There is evidence of a high genetic barrier to developing resistance against remdesivir in coronavirus, because of which it maintains its effectiveness in antiviral therapies against these viruses.^12^

It has shown in vitro activity on human airway epithelial cells against SARS-CoV-2. Remdesivir has previously been used in treating the Ebola virus.^13^ In the US, the first COVID-19 patient was treated with remdesivir, with significant improvement within 24 h of the treatment.^14^ On May 1, 2020, this investigational drug was granted an emergency use authorization by the food and drug administration (FDA).^15^

Remdesivir has shown benefit in hospitalized SARS-CoV-2 patients with pneumonia with improved clinical outcomes, shorter time to recovery, reduced hospital stays and lower mortality.^3^^,^^16^ Remdesivir is also associated with a decrease in viral load when administered in the initial disease process.^17^

This study evaluates the clinical effectiveness, reduction in disease severity, duration of hospital stays of the patients, and clinical outcome of COVID-19 patients admitted to the hospital who received remdesivir.

## 2 Materials and methods

This was a Quasi-experimental single-center study aiming at the estimation of the causal impact of remdesivir. The study was conducted at Lahore General Hospital (LGH), Lahore, Pakistan. L.G.H. is a 1600 bed tertiary care hospital. Out of 1600 beds, 95 were allocated for COVID-19 patients. These beds were of varying acuity from the medical floor, to Intensive Care Units (ICUs) and High Dependency Units (HDUs).

### 2.1 Ethical consideration

Ethical approval was obtained from the Research Ethical Committee of Lahore General Hospital, Lahore, having approval number: 00–143-20. The study was conducted in full conformance with principles of the “Declaration of Helsinki,” Good Clinical Practice (GCP), and within the laws and regulations of the Pakistan Health Research Council (PHRC).

### 2.2 Participants

846 suspected COVID-19 patients admitted to the hospital were screened by nasopharyngeal RT-PCR for SARS-CoV-2. 612 SARS-CoV-2 nasopharyngeal RT-PCR positive patients were evaluated for enrollment in the study. A total of 159 patients received remdesivir.

**Figure:**
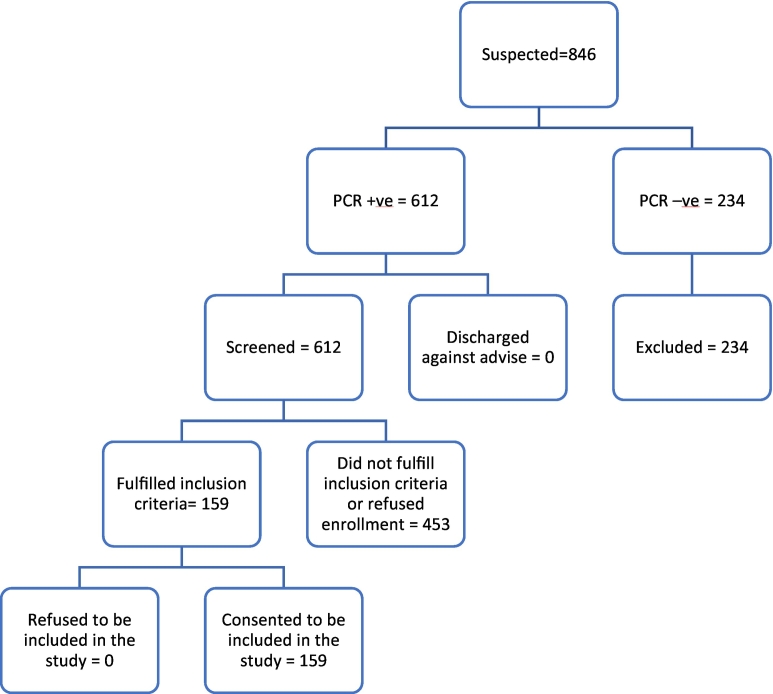

### 2.3 Inclusion criteria

• Age 15–75 years
• COVID-19 diagnosed by a nasopharyngeal RT-PCR.
• COVID-19 illness requiring hospitalization
• Respiratory rate > 22/ min
• Patients having classical radiological lesions of COVID-19 on X-ray chest or HRCT chest.
• COVID-19 patients who failed to respond to therapy. The failure to therapy was as defined by either clinical deterioration or worsening of the laboratory findings or worsening radiological findings post local standard of care (antibiotics, methylprednisolone, and enoxaparin sodium) as determined by the treating clinician i.e.
  o Persisting tachypnea beyond 22/min
  o An increase in O2 requirement,
  o ≥2 biochemical markers (Lymphocyte count, C-Reactive Protein CRP, Lactate dehydrogenase LDH, D-dimer, Serum Ferritin) are increasing by >30%
  o More than 50% radiological deterioration.

### 2.4 Exclusion criteria

• Patients on Invasive mechanical ventilation (IMV).
• History of allergy to remdesivir or any of its components
• Presences of chronic renal failure >4 stage, GFR < 30 ml/min/1.73m^2^.
• ALT/AST > 5 times than normal values.
• Pregnant women.

### 2.5 Variables

The information collected included patients' demographics, presenting symptoms, disease severity, comorbidities, including the history of smoking, hypertension, diabetes mellitus, asthma, chronic obstructive pulmonary disease, tuberculosis, hepatitis, and chronic kidney disease. (see attached form in Appendix.) Home medications were documented. Laboratory tests in which complete blood count (CBC), liver function tests (LFTs), renal function tests (RFTs), C-reactive protein (CRP), lactate dehydrogenase (LDH), serum ferritin, D-dimer were recorded on the 1st, third day, and seventh day of treatment. LFTs and RFTs were monitored before initiating remdesivir. Vital signs were recorded both pre-remdesivir and post-remdesivir intervention. The dose of the drug, its duration, and outcomes, including the length of hospital stay, complications, and mortality of patients after remdesivir intervention, were abstracted on the approved case record form. (Appendix 1).

### 2.6 Remdesvir intervention

A loading dose of 200 mg Intravenous (IV) remdesivir was given to moderate disease severity patients of COVID-19. This was followed by the maintenance dose of 100 mg IV for a minimum of 5 days and a maximum of 10 days.

### 2.7 Data analysis

Data was collected from the hospital's electronic medical record. Each patient's data was abstracted into a case record form (CRF) and was subsequently accessed by study investigators before being exported into a study-specific excel spreadsheet. Each entry was coded using a unique patient identifier, accessible only to investigators. Links between the names, and the unique patient identifier codes were kept in an Excel sheet locked within the principal investigator's (PI) computer and was destroyed post completion of analysis.

Data was analyzed by S.P.S.S. version 25. Pearson Chi-square test was applied to determine the independence of variables. This was followed by applying correlation analysis to determine the effect of early administration of the drug on the outcome of the disease. Case Fatality Ratio (CFR) was compared among COVID-19 patients who received remdesivir and those who did not, as it may reflect the efficiency of treatment. The graphs for the fluctuations in laboratory findings at Day 1 and Day 7 were created by Microsoft Visual Studio 2019 version 16.8.3.

## 3 Results

A total of 159 COVID-19 patients were received remdesivir. The majority were males (60%, *n* = 96), and the majority (48%, *n* = 76) ranged between 46 and 60 years of age. The mean age was 47.54 S. D ± 12.70 The patients were categorized as having symptoms of moderate disease according to guidelines. (Table 1).

**Table 1:** Demographics and symptomatic distribution of Mild, Moderate, and severe illness of COVID-19 patients.

Variable	N = 159
Gender	
Male	96 (60%)
Female	63 (40%)
Age	
15–30	18 (11%)
31–45	41 (26%)
46–60	76 (48%)
61–75	24 (15%)
Disease severity	
Low-grade fever	12 (7.5%)
Cough	94 (59%)
Malaise	103 (65%)
Rhinorrhea	6 (4%)
Loss of sense of taste	74 (46.5%)
Loss of sense of smell	74 (46.5%)
High-grade fever	36 (22.6%)
G.I. symptoms	32 (20%)
Confusion and lethargy	26 (16%)
Comorbidities	
Smoking	26 (16%)
Diabetes mellitus	41 (26%)
Hypertension	61 (39%)
Asthma	65 (41%)
Tuberculosis	8 (5%)
Hepatitis B/C	5 (3%)
Other comorbidities	17 (11%)

The percentage of lungs involvement in Chest X-Ray (CXR) of the patients of COVID-19 was; 9 patients (6%) had <25% unilateral involvement of lungs in CXR, 84 patients (53%) had <25% bilateral involvement of lungs, 35 patients (22%) had 25–50% bilateral involvement of lungs and 31 patients (19%) had >50% bilateral involvement of lungs.

The mean (± SD) of the day at which the first dose of remdesivir was administered was 5.80 (± 2.59) for the day at which 1st dose of remdesivir was administered. (Table 2,Table 3.)

**Table 2:** Distribution of remdesivir intervention according to days on which the first dose is administered and its outcome.

Day on which ^the first^ dose of remdesivir administered	OUTCOME		
	Recovered	Died	Total
1–3 Days	24 (15%)	0 (0%)	24 (15%)
4–6 Days	70 (44%)	7 (4.5%)	77 (48.5%)
7–9 Days	35 (22%)	4 (2.5%)	39 (24.5%)
10–12 Days	12 (7.5%)	7 (4.5%)	19 (12%)
Total	142 (88.5%)	18 (11.5%)	159 (100%)

**Table 3:** Distribution of outcome of remdesivir administration according to the dose of the antiviral drug.

No of doses	Recovered	Died	Total
Less than 5 doses	18 (11%)	4 (2.5%)	22 (14%)
5 doses	84 (53%)	8 (5%)	92 (58%)
6–10 Doses	39 (24.5%)	6 (4%)	45 (28%)
Total	141 (88.5%)	18 (11.5%)	159 (100%)

The mean (± SD) number of doses of remdesivir administered to the patients was 5.42 (± 1.47) for number of doses of remdesivir administered. The chi-square of independence was performed to examine the relation between the day the first dose was administered, the remdesivir dose, and the outcome. The relation between these variables was significant. For day on which 1st dose was administered and outcome *X*^*2*^ *(3, N* *=* *159) 51.994, p* ≤ *0.001*. For remdesivir dosage and outcome *X*^*2*^ *(2, N* *= 159) 48.038, p* ≤ *0.001*. Both the early administration and the remdesivir dosage showed significant association with the positive outcome. (Table 4).

**Table 4:** Association between remdesivir first dose administration, No of doses, and its outcome.

	Day on which 1st Dose was Administered	Remdesvir Dose	Remdesvir (Outcome)
Pearson Chi-Square	51.994	48.038	95.151
Df	3	2	1
Asymp. Sig.	0.000	0.000	0.000

Among 159 patients who received remdesivir, the day on which the first remdesivir dose was administered was found to be significantly correlated, with the prognosis of the disease in the patients, on applying the Pearson correlation test, r (1) = 0.273, *p* < .001. The positive value of Pearson correlation showed that the earlier administration of remdesivir is related to a better outcome in patients, as shown in Table 5. The mean (± SD) duration of stay in the hospital was found to be 8.46 days (± 3.278) for the duration of stay in hospital.

**Table 5:** Regression Analysis of Various Drug Factors based on Patient Mortality.

ANOVA						
Model		Sum of squares	Df	Mean square	F	Sig.
1	Regression	8.005	5	1.601	4.387	0.001
	Residual	55.844	153	0.365		
	Total	63.849	158			

a. Dependent Variable: Remdesvir (Outcome).

b. Predictors: (Constant), Remdesvir (No of Doses), Gender, Day on which 1st Dose was Administered, Age, Remdesvir (Duration of Hospital Stay).

**Fig. 1:**
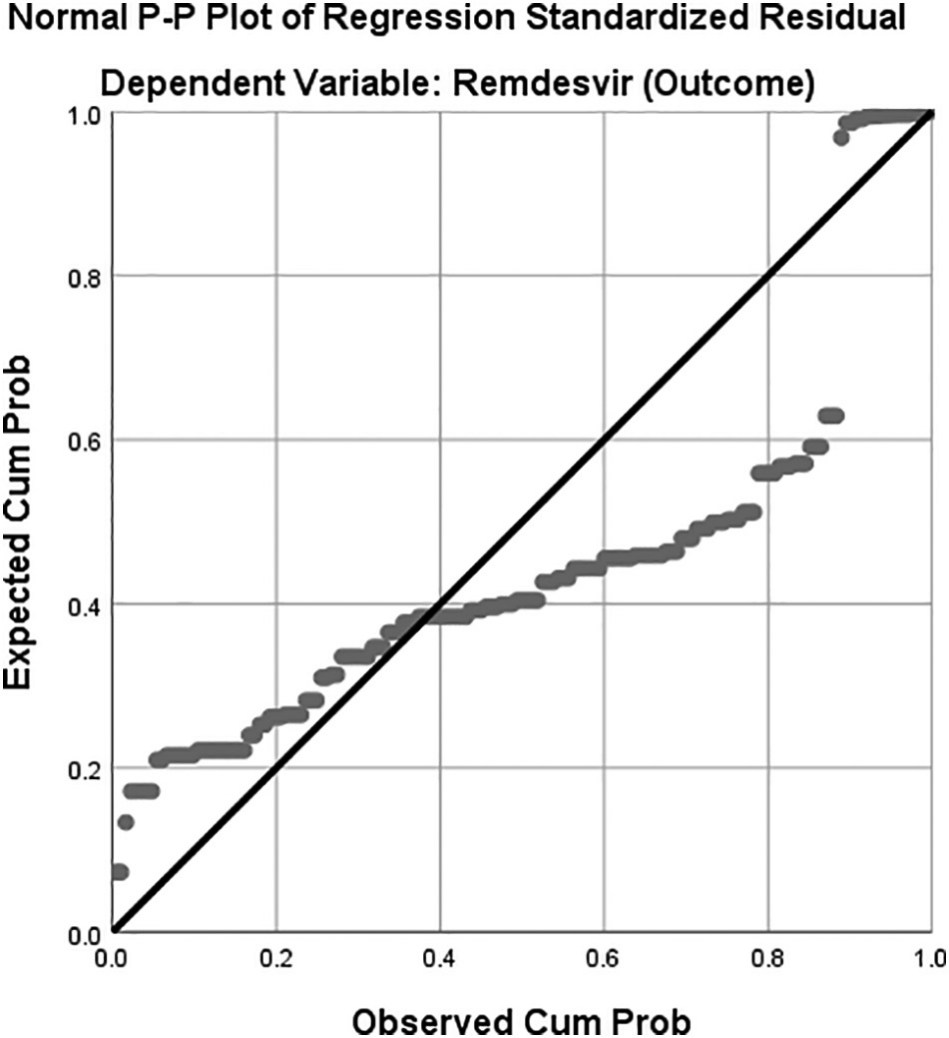
Graphical representation of the Regression analysis by NORMPROB (ZRESID).

**Fig. 2:**
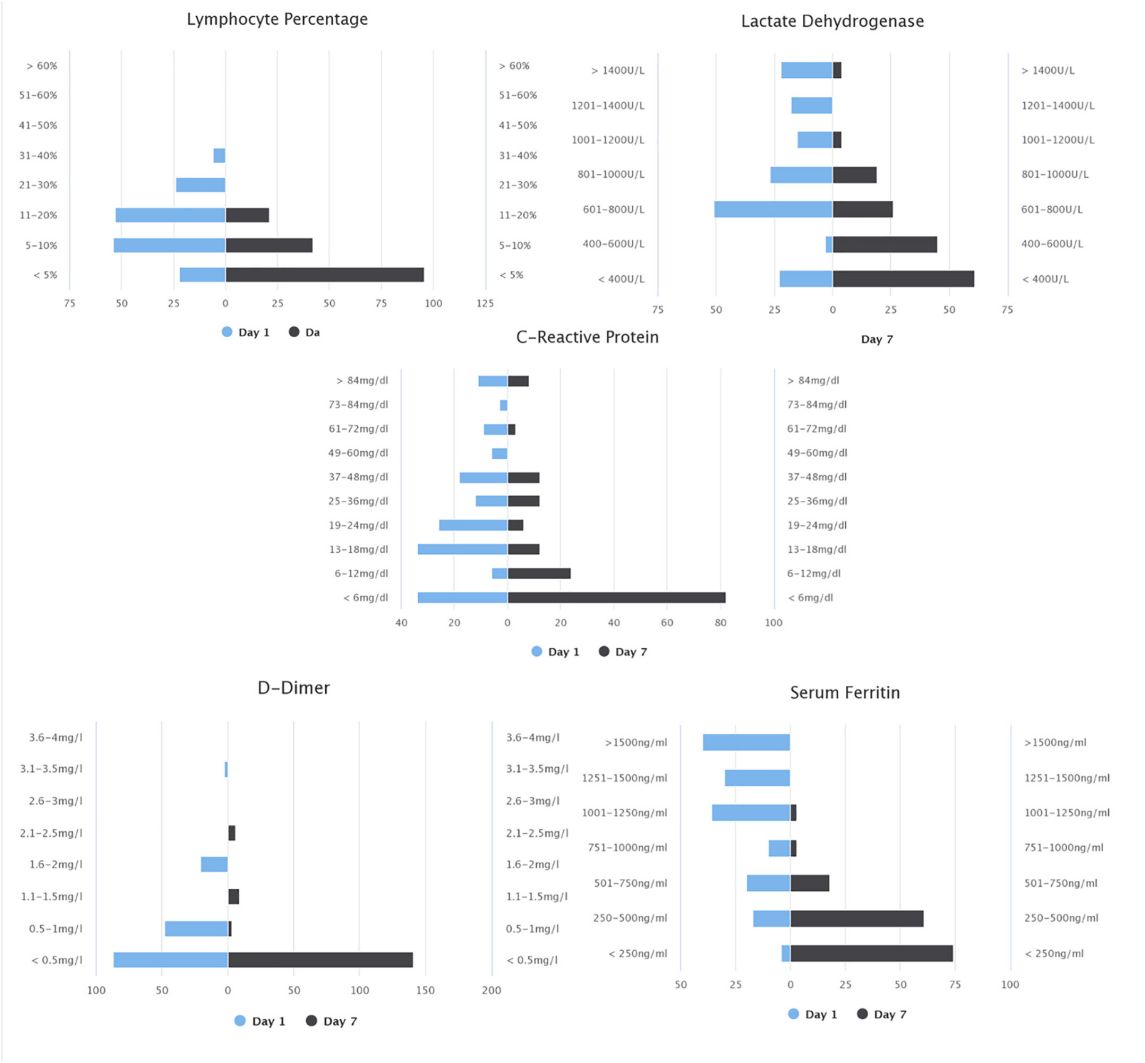
Laboratory findings of Lymphocyte % age, C-reactive protein, D-dimer, and serum ferritin level of 159 COVID-19 patients receiving remdesivir on day 1 of treatment and the 7th day of treatment.

There was a statistically significant difference between groups as determined by one-way ANOVA (*F*(5,153) = 4.387, *p* = .001). The NORMPROB was plotted to assess whether or not our data is approximately normally distributed. Although a maximum of the sample was distributed normally but some variance from the normal was observed as evident from the plot (Fig. 1). (See Fig. 2.)

The standardized case fatality ratio (CFR) was in the 453 hospitalized patients who did not receive remdesivir was 32.89% (*n* = 149). In 159 Patients who received remdesivir, the standardized CFR dropped down to 11.32% (*N* = 18), proving the likely therapeutic value of remdesivir.

## 4 Discussion

The treatment guidelines for COVID-19 have been evolving since the start of the pandemic, and there is a global lack of conformity in the management strategies.^17^ In this study, out of 612 SARS-CoV-2 nasopharyngeal RT-PCR positive patients, 159 patients were selected by convenient sampling, who fulfilled the inclusion and exclusion criteria.

Previous data show males are relatively more susceptible to SARS-CoV-2 infection. Males presented with more severe disease, and their mortality is double compared to females, which is also consistent with this study^18^^,^^19^ having high-grade fever and oxygen saturation < 93%. Similarly, fever was documented in 72–98% as the most frequent symptom. A decrease in oxygen saturation is a predictor of severity of COVID-19.20., 21, 22 The most common comorbid condition was diabetes mellitus which is also well-documented.^23^^,^^24^

Remdesivir was given as an adjuvant therapy along with combination of antibiotics, methylprednisolone, and enoxaparin sodium to COVID-19 patients in this center. 159 COVID-19 patients included in the study received remdesivir as they failed to respond to the drug combination of ‘antibiotics, methylprednisolone and enoxaparin sodium’. Most of the COVID-19 patients showed bilateral lung involvement in chest X-rays; these findings are consistent with the study conducted by Durrani et al.^25^ Better outcome was observed in this study when treated with remdesivir. Early intervention of remdesivir may account for better prognosis in moderate disease. Most of patients in this study had moderate disease, and 96.4% of the patient recovered from moderate illness, while only one patient died in moderate disease after receiving remdesivir.

Most of the patients received the first dose of remdesivir at 4–6 days of admission, i.e., the early phase of the illness. It significantly reduced the duration of hospital stay from a range of 15–21 days, in patients not receiving remdesivir, to less than 14 days, in patients who received remdesivir. Among 159 patients, 43 patients were discharged in less than seven days, 109 patients in 7–14 days, and seven patients in 15–21 days. Beigel et al. supported these results with their more extensive study (*N* = 1026). They also documented the reduction of recovery time and a hospital stay of COVID-19 patients compared to the placebo group of the study.^26^ A study by Wang et al. also revealed that remdesivir outcome was good in patients who were administered the drug within ten days of symptoms of the disease compared to those who received treatment in later days.^11^

In the results of this study, 13.8% (*N* = 22) of patients received less than 5, 57.9% (*N* = 92) of the patients received five doses course of remdesivir, and 28.3% (*N* = 45) of the patients received 6–10 days course of remdesivir inclusive of the one loading dose. They recovered and showed a good prognosis of the disease. Earlier literature showed ten days of treatment with remdesivir attained good clinical improvement compared to five days.^27^ COVID-19 patients with Oxygen saturation < 94%, receiving remdesivir led to an improvement in the clinical condition of 68% of patients.^27^ Among 159 patients, 82.4% (*N* = 131) had oxygen saturation < 94% out of which <86.25% (*N* = 113) showed complete recovery when treated with remdesivir. In the results, the negative value of Pearson correlation showed that early administration of remdesivir is related to improvement in oxygen saturation.

In this study, the multivariate regression analysis showed a significant relationship between the outcome of disease and its predictors i.e., age, gender, day on first dose of remdesivir is administered, number of doses of remdesivir given and duration of hospital stay of the patients.

The laboratory investigations of this study showed that CRP, D-dimers, LDH, Serum ferritin declined on the 7th day of treatment. However, leukopenia persisted beyond seven days. Most of the patients, 60% (*N* = 96), had lymphocytes count <5%. Tan et al. reported lymphocytes <5% for 15 days after disease onset and suggested it as a marker for disease severity.^28^ We observed derangement of LFTs after remdesivir intervention, which is also documented in literature.^29^

The primary outcome of this study was clinical effectiveness, reduction in disease severity, reduced duration of hospital stays of the patients, and clinical outcome of COVID-19 patients with remdesivir. It was observed in this study that after the failure of combination therapy of antibiotics, methylprednisolone and enoxaparin sodium, remdesivir played an essential role as an adjuvant by supporting the basic treatment, and disease severity of COVID-19 disease.

The main limitations of this study are: a relatively small sample size derived from convenient sampling, non-blinded design, an absence of a case-control design and a short follow up period.

## 5 Conclusion

Remdesivir is associated with decreasing the severity of the SARS-CoV-2 disease in COVID-19 patients. It has also decreased the patients' hospital stay and improved the clinical outcome and mortality of the patients.

## Funding body

No funding body in this study.

## Consent

Informed consent was obtained from all the patients who agreed to publish their data in this research. We had protected patient privacy and obeyed the Helsinki Declaration. Patients who fulfilled the inclusion criteria were enrolled. No side effects were observed during the clinical trial of remdesivir, but in a few patients prolonged steroid-induced lymphopenia was observed. We observed derangement of LFTs after remdesivir intervention.

## Declaration of Competing Interest

No conflict of interest among the authors.

## Appendix A Supplementary data

Supplementary material Image 1
